# Role of the LAMMER kinase LkhA in fungal development and aflatoxin production in *Aspergillus flavus*

**DOI:** 10.71150/jm.2503007

**Published:** 2025-05-27

**Authors:** Seong-Hwan Jeong, He-Jin Cho, Jae-Hyuk Yu, Hee-Moon Park, Hee-Soo Park

**Affiliations:** 1 Department of Integrative Biology, Kyungpook National University, Daegu 41566, Republic of Korea; 2 School of Food Science and Biotechnology, Kyungpook National University, Daegu 41566, Republic of Korea; 3 Department of Bacteriology, University of Wisconsin-Madison, Madison, Wisconsin 53706, USA; 4 Department of Microbiology and Molecular Biology, College of Bioscience and Biotechnology, Chungnam National University, Daejeon 34134, Republic of Korea; 5 Department of Advanced Bioconvergence, Kyungpook National University, Daegu 41566, Republic of Korea

**Keywords:** *Aspergillus flavus*, aflatoxin B1, LAMMER kinase, pathogenicity

## Abstract

A well-conserved LAMMER kinase in yeast and filamentous fungi, is a dual-specificity kinase with multiple roles in fungal biology. In this study, we assessed the roles of LkhA in *Aspergillus flavus*, a toxigenic fungus that produces aflatoxin B1. *lkhA* deletion mutants exhibited defects in fungal growth, conidiophore development, and sclerotia formation. These mutants exhibited impaired tolerance to oxidative and cell wall stresses. Moreover, the absence of *lkhA* resulted in a decrease in aflatoxin B1 production. The kernel assay revealed that the *lkhA* deletion mutants exhibited reduced production of conidia and aflatoxin B1, implying that LkhA can affect fungal toxigenesis and pathogenicity. Taken together, these results demonstrate that LkhA is important for differentiation, mycotoxin production, and pathogenicity in *A. flavus*.

## Introduction

*Aspergillus flavus* is a pathogenic fungus that infects peanuts and corn and causes economic losses (Amaike and Keller, 2011; Fountain et al., 2014; Hedayati et al., 2007). This fungus produces aflatoxin B1, a class 1 carcinogen, in crops (Klich, 2007; Kumar et al., 2021). Contamination by aflatoxins produced by *A. flavus* has been reported to result in harvest losses worth millions of dollars from crops like corn and peanuts (Amaike and Keller, 2011). Moreover, *A. flavus* is an opportunistic fungus in animals and the second most common cause of aspergillosis in immunocompromised humans (Krishnan et al., 2009; Lim and Park, 2019; Pasqualotto, 2009).

*A. flavus* is known to reproduce asexually or sexually; it primarily reproduces asexually (Horn et al., 2009; Krijgsheld et al., 2013; Ojeda-López et al., 2018). The asexual reproduction of *A. flavus* results in the production of asexual spores called conidia, which are elaborately regulated by BrlA–AbaA–WetA, transcription factors involved in a central regulatory cascade (Cho et al., 2022; Park and Yu, 2012a). The formed conidia spread in the air and regulate the onset of germination depending on the environment (Baltussen et al., 2020; Wang et al., 2021). The asexual reproductive cycle of *A. flavus* begins with the germination of conidia, followed by the formation of new conidia (Baltussen et al., 2020; Park and Yu, 2012a). Sexual reproduction results in the formation of sexual structures called sclerotia under certain conditions (Dyer and O'Gorman, 2012; Horn et al., 2016). Sclerotia contain sexual spores and help protect them from extreme environments (Dyer and O'Gorman, 2012). Asexual or sexual spores remain dormant when *A. flavus* is in an environment unfavorable for its survival; they germinate again when the environment improves (Coley-Smith and Cooke, 1971; Wicklow, 1987). Both asexual and sexual forms of development are induced by specific conditions and regulated by various signaling mechanisms (Park et al., 2019; Park and Yu, 2016). Stress resistance and secondary metabolism are the results of adaptation to various environments and are associated with kinase-related signaling pathways (Lin et al., 2021; Zhao et al., 2007).

The dual-specificity LAMMER kinases are evolutionarily conserved across diverse species ranging from yeast to mammals and have multiple functions in various physiological processes (Kang et al., 2013; Lim et al., 2021; Lim and Park, 2019). The LAMMER kinase family is essential for the phosphorylation of serine/threonine and tyrosine residues (Lim and Park, 2019; Yun et al., 1994). The name “LAMMER kinase” is derived from the “EHLAMMERILG” motif in subdomain X (Duan et al., 2016; Yun et al., 1994). The LAMMER kinase motif is important for substrate recognition, activity, and localization (Kang et al., 2010; Savaldi-Goldstein et al., 2003). LAMMER kinases play essential roles in mammals, with deficiency mutations causing various diseases, such as cancer (Chowdhury et al., 2023). In fungi, LAMMER kinases are involved in various biological processes (Lim and Park, 2019). LkhA plays a role in hyphal growth, sexual reproduction, and asexual reproduction in *A. nidulans* (Kang et al., 2013). In *A. fumigatus*, LkhA is involved in sexual and asexual reproduction, causing changes in gliotoxin production and cell wall composition. It also affects the pathogenicity of *A. fumigatus* (Lim et al., 2021; Lim and Park, 2019). However, the role of LAMMER kinases has not yet been studied in *A. flavus*.

Therefore, in this study, we assessed the role of the LAMMER kinase LkhA in the toxin-producing fungus *A. flavus*. Based on the results obtained in other fungal species, such as *A. fumigatus* and *A. nidulans*, we hypothesized that this LAMMER kinase plays a role in cell growth and toxin production in *A. flavus*. To test this hypothesis, we generated the *lkhA* deletion mutant (Δ*lkhA* strain) and assessed its phenotype.

## Materials and Methods

### Phylogenetic analysis

The LAMMER kinase protein sequences of *A. nidulans* FGSC A4, *A. fumigatus* Af293, and *Aspergillus* section *Flavi* were downloaded from FungiDB (https://fungidb.org/). A phylogenetic tree of LAMMER kinase orthologs was generated using MEGA7 software with the maximum likelihood method based on the Jones-Taylor-Thornton (JTT) matrix-based model (http://www.megasoftware.net/). A bootstrap consensus tree was inferred from 1,000 replicates, and the taxa in this tree were assumed to represent evolutionary history. The domain was analyzed using NCBI’s Conserved Domain Database (CDD) under the ID cd14134.

### Strains, media, and culture conditions

The fungal strains used in this study are listed in Table 1. *A. flavus* strains were cultured at 30°C or 37°C in glucose minimal medium containing 0.1% yeast extract (MMYE) with the appropriate supplements (Barratt et al., 1965). *A. flavus* NRRL 3357.5, a *pyrG*^−^ auxotrophic mutant strain, was grown on MMYE supplemented with uridine and uracil (He et al., 2007). For transformation, *Escherichia coli* DH5α cells were grown in Luria-Bertani (LB) broth with or without ampicillin.

### Construction of deletion mutant and complemented strains

The primers used to construct deletion mutant and complemented strains are listed in Table 2. Gene deletion cassettes were generated using double-joint PCR (DJ-PCR) (Yu et al., 2004). Initially, the 5′ and 3′ flanking regions were amplified from *A. flavus* NRRL 3357 genomic DNA (gDNA) using the primer sets OHS2349:OHS2350 and OHS2351:OHS2352, respectively. The *A. fumigatus* *pyrG*^+^ marker was amplified from *A. fumigatus* AF293 using OHS1542:OHS1543. The products and the marker were re-amplified using OHS2353:OSH2354 to generate the *lkhA* deletion cassette. This cassette was transformed into the protoplast of *A. flavus* NRRL 3357.5. Protoplasts were generated using VinoTaste^®^ Pro (Novozymes, Denmark) (Lee et al., 2016). Transformation candidates were confirmed by performing PCR, restriction enzyme treatment, and quantitative reverse-transcription PCR (qRT-PCR). Three independent Δ*lkhA* strains (TJSH4.1–3) were isolated.

To generate the complemented strain, the promoter and ORF of *lkhA* were amplified using OHS2565:OHS2405. The PCR product was digested with NotⅠ, and the digested product was then cloned into pYES1, including the 4× FLAG tag, *ptrA*, and the *amyB* terminator. The resulting plasmid, pJSH1, was transformed into TJSH4.1. Final complemented strains (TJSH5.1 and 2) were verified by performing PCR and qRT-PCR.

### qRT-PCR

RNA samples were obtained using a previously described protocol (Park et al., 2012; Park and Yu, 2012b). In brief, conidia of control and mutant stains were inoculated in liquid MMYE media and cultured at 37°C with shaking at 200 rpm for 16 h. The grown mycelia were filtered through Miracloth (Calbiochem, USA), washed with desterilized water, and spread in a monolayer on solid MMYE to induce asexual development. Samples for RNA extraction were obtained at each designated time (Eom et al., 2018). The samples were mixed with TRIzol reagent (Invitrogen, USA), homogenized using a mini-bead beater, and centrifuged to obtain the supernatant. The supernatant was transferred into a new vial in the presence of cold isopropanol. DNase I (Promega, USA) was added to each sample to remove DNA contamination. The purity and concentration of RNA were measured using an ultraviolet (UV) spectrophotometer. Complementary DNA (cDNA) was synthesized using GoScript reverse transcriptase (Promega, USA). qPCR was performed using the iTaq Universal SYBR Green Supermix (Bio-Rad, USA) and the CFX96 Touch Real-Time PCR System (Bio-Rad). The β-actin gene was used as a control. All experiments were performed in triplicate. The primers used for qPCR are listed in Table 2.

### Physiological analyses

To analyze asexual development, control, deletion mutant, and complemented strains were inoculated onto solid MMYE media and incubated for 5 days at 37°C in light. The diameter of the colony of each strain was then measured to assess fungal growth. To measure the number of conidia, conidia were collected from 5-day-old culture plates and counted using a hemocytometer.

To assess the formation of sclerotia, control or mutant strains were inoculated onto solid MMYE agar and incubated at 37°C for 7 days in darkness. To assess the formation of sclerotia, the cultured plates were washed with 70% ethanol and the number of sclerotia was then counted. All experiments were performed in triplicate.

Photographs of each strain were taken using a Pentax MX-1 digital camera, and micrographs were taken using a Zeiss Lab.A1 microscope equipped with AxioCam 105 and AxioVision (Rel. 4.9) digital imaging software.

### Stress sensitivity test

To determine whether stress affects fungal growth, control, deletion mutant, and complemented strains were inoculated onto solid MMYE medium containing Congo red (cell wall stressor) or hydrogen peroxide (H_2_O_2_) (oxidative stressor) and incubated at 37°C for 3 days. The inhibition rate was determined by calculating the area based on the diameter. The experiment was performed in triplicate for each strain.

### Aflatoxin B1 extraction and thin-layer chromatography (TLC)

To extract aflatoxin B1 each strain was inoculated into complete medium (CM) and incubated at 30°C for 7 days (Eom et al., 2018). Subsequently, CHCl_3_ was added to extract aflatoxin B1 from the grown cultures. Each sample was mixed using Voltexer, and the organic phase was separated by centrifugation. The organic phase was then filtered through Whatman filter paper and treated with Na_2_SO_4_. The samples and aflatoxin B1 as standard were spotted onto TLC silica plates (Kieselgel 60, 0.25 mm, Merck KGaA, Germany). A chloroform:acetone solution (8:2 ratio) was used for saturation in the chamber to separate the mixture. To detect aflatoxin B1, the plate containing the samples was examined under 366 nm UV light irradiation. Aflatoxin B1 band intensity was quantified using ImageJ software. The experiment was performed in triplicate for each strain.

### Kernel bioassay

Corn kernels (Cheongnong, Korea) were treated with 70% ethanol, washed with ddH_2_O, and treated with bleach thrice. Then, the kernels were placed on a plate, inoculated with each strain, and incubated at 30°C for 7 days. Conidia from each kernel were obtained using 2 ml of 0.01% Tween 20, and the number of conidia was counted using a hemocytometer. To extract aflatoxin B1, kernels were mixed with CHCl_3_ for 4 h. The conidia were then crushed with a bead beater (BioSpec Products Inc., USA), and the aflatoxin B1 was extracted using a previously described method (Christensen et al., 2012).

### Statistical analysis

Statistical analysis was performed using GraphPad Prism (version 5.01). Statistical differences between strains were assessed using Student’s unpaired t-test, with error bars representing standard errors of the means of triplicate measurements. Moreover, *p* values < 0.05 were considered to indicate significant differences between control and deletion mutant strains (*p ≤ 0.05, **p ≤ 0.01, ***p ≤ 0.001).

## Results

### Domain analysis of LkhA in *A. flavus*

**Fig. 1.**
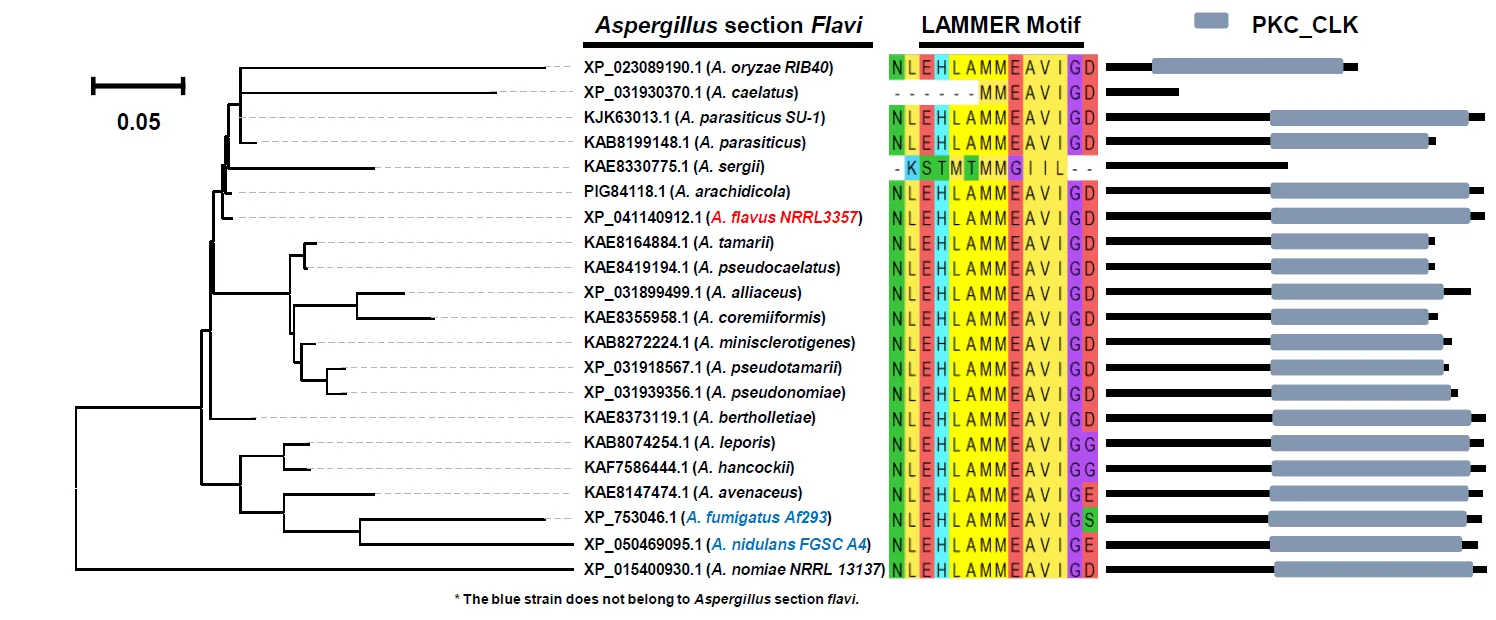
Domain analysis of LkhA. A phylogenetic tree of LkhA homologs of 19 *Aspergillus* section *Flavi* species, including XP_023089190.1 (*A. oryzae* RIB40), XP_031930370.1 (*A. caelatus*), KJK63013.1 (*A. parasiticus* SU-1), KAB8199148.1 (*A. parasiticus*), KAE8330775.1 (*A. sergii*), PIG84118.1 (*A. arachidicola*), XP_041140912.1 (*A. flavus* NRRL 3357), KAE8164884.1 (*A. tamarii*), KAE8419194.1 (*A. pseudocaelatus*), XP_031899499.1 (*A. alliaceus*), KAE8355958.1 (*A. coremiiformis*), KAB8272224.1 (*A. minisclerotigenes*), XP_031918567.1 (*A. pseudotamarii*), XP_031939356.1 (*A. pseudonomiae*), KAE8373119.1 (*A. bertholletiae*), KAB8074254.1 (*A. leporis*), KAF7586444.1 (*A. hacockii*), KAE8147474.1 (*A. avenaceus*), XP.753046.1 (*A. fumigatus* Af293), XP_0540469095.1 (*A. nidulans* FGSC A4), and XP_015400930.1 (*A. nomiae* NRRL 13137). Conservation of the LAMMER motif was analyzed using MEGA7 and the NCBI database.

Recent studies have analyzed the domains of LkhA in two *Aspergillus* species: *A. nidulans* and *A. fumigatus*. XP_041140912.1, a LAMMER kinase from *A. flavus*, was identified using “EHLAMMERILG,” a conserved motif of known LAMMER kinases from *A. nidulans* and *A. fumigatus* (Kang et al., 2013; Lim et al., 2021; Lim and Park, 2019). Based on XP_041140912.1, homology was confirmed with *Aspergillus* section *Flavi*, *A. nidulans*, and *A. fumigatus*. In the NCBI database, LkhA could only be identified in 19 species of *Aspergillus* section *Flavi*. Therefore, we analyzed the phylogenetic tree and domain of LkhA in *A. nidulans*, *A. fumigatus*, and 19 species of *Aspergillus* section *Flavi*. Our results suggest that XP_041140912.1 encodes the LkhA homolog in *A. flavus* (Fig. 1).

### Role of LkhA in asexual development

**Fig. 2.**
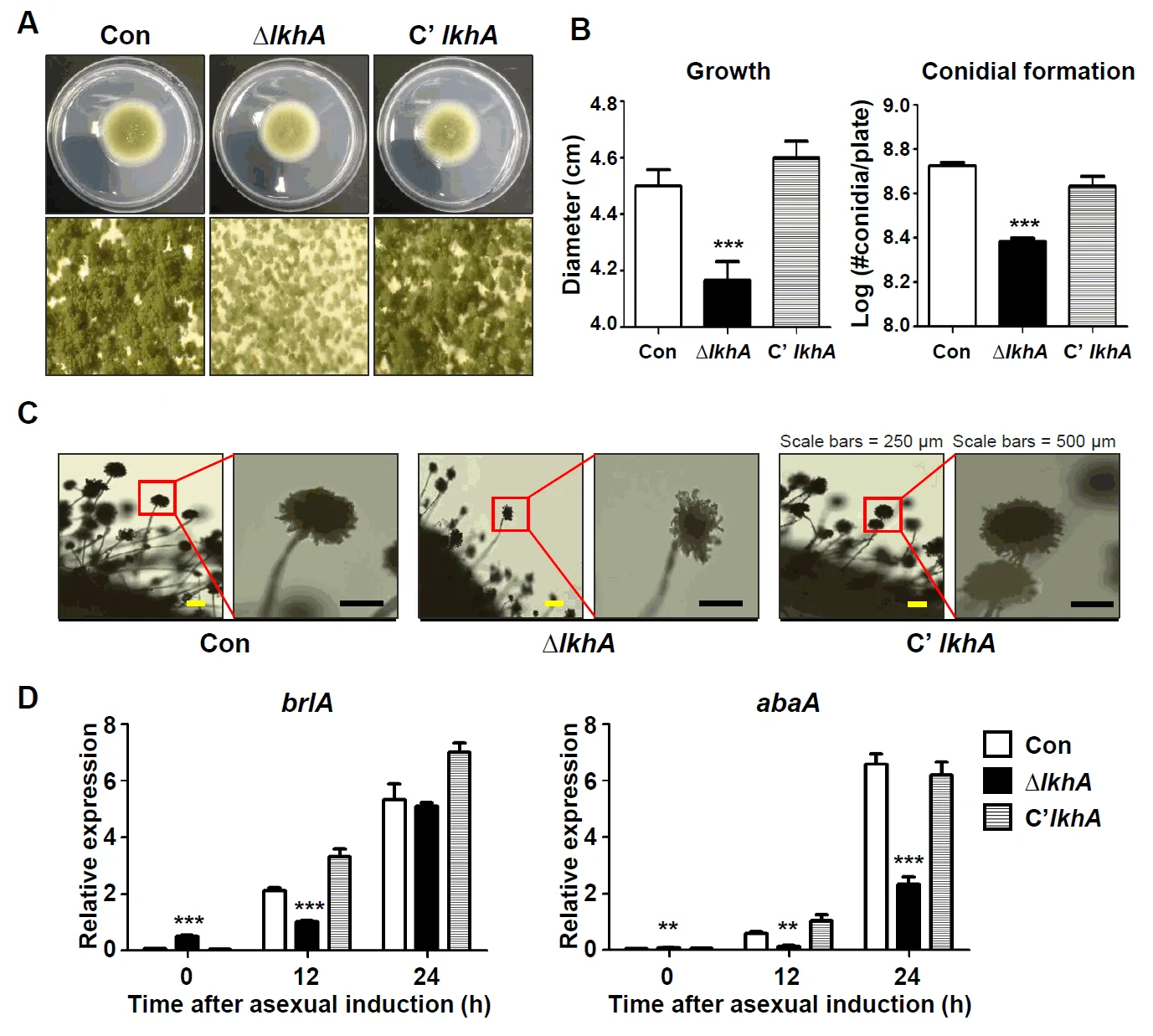
Roles of LkhA in fungal growth and asexual development. (A) Photographs and micrographs of control, *lkhA* deletion mutant, and *lkhA* complemented strains cultured for 5 days in light. (B) Diameter and number of conidia measured in the presence of light. (C) Appearance of conidiophores grown in light 2 days after inoculation. (D) The mRNA expression levels of *brlA* and *abaA*, which are key regulatory genes for asexual development, were analyzed in control, *lkhA* deletion mutant, and *lkhA* complemented strains by performing qRT-PCR. RNA samples from each strain were used at 0, 12, and 24 h after the induction of asexual development. Error bars represent the standard errors of the means from triplicate measurements (Control versus Δ*lkhA* strain, ^**^p ≤ 0.01, ^***^p ≤ 0.001).

As LkhA plays a role in asexual reproduction in *A. nidulans* (Kang et al., 2013), we hypothesized that LkhA is also involved in asexual reproduction in *A. flavus*. To test this hypothesis, we generated a Δ*lkhA* strain and a *lkhA* complemented strain (*C' lkhA* strain) and performed phenotypic analysis (Fig. 2). We found that the colony diameter and number of conidia in the Δ*lkhA* strain were lower than those in the control strain in the presence of light (Fig. 2B). Microscopic analysis revealed that the Δ*lkhA* strain formed abnormal conidiophores (Fig. 2C). The mRNA expression of *brlA* in the Δ*lkhA* strain decreased 12 h after the induction of conidiation. Moreover, the mRNA expression of *abaA* in the Δ*lkhA* strain was lower than that in the control or *C' lkhA* strain at 12 and 24 h after the induction of asexual development (Fig. 2D). These results suggest that LkhA is essential for proper asexual development in *A. flavus*.

### Role of LkhA in sexual development

**Fig. 3.**
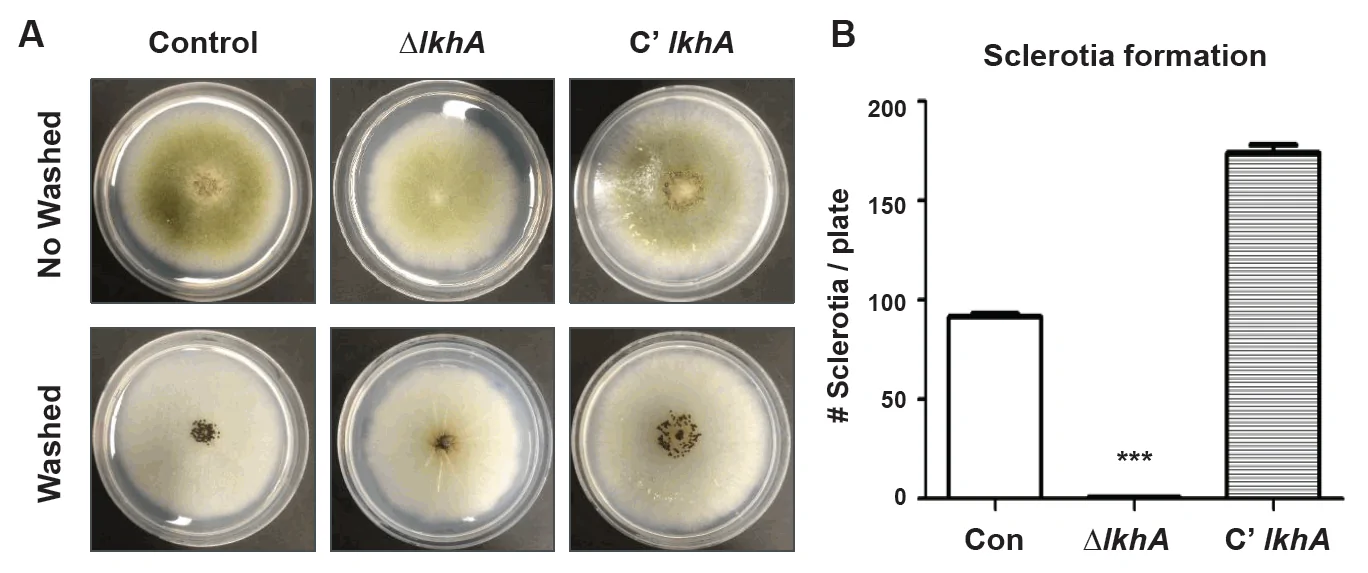
Roles of LkhA in sexual development. (A) Photographs of control, *lkhA* deletion mutant, and *lkhA* complemented strains cultured for 7 days in darkness. Bottom: Photographs of control, *lkhA* deletion mutant, and *lkhA* complemented strains after ethanol washing. (B) Quantitative data of sclerotia, indicative of sexual reproduction (^***^p ≤ 0.001).

LkhA plays a role in sexual development in *A. nidulans* and *A. fumigatus* (Kang et al., 2013; Lim et al., 2021). Therefore, we assessed the role of LkhA in sexual reproduction in *A. flavus*. We inoculated each strain onto solid MMYE medium and incubated it for 7 days in darkness. We then washed the culture with alcohol and counted the number of sclerotia (Fig. 3A). We did not detect normal sclerotia in the Δ*lkhA* strain (Fig. 3A). The number of sclerotia in the Δ*lkhA* strain was dramatically lower than that in the control or *C' lkhA* strain (Fig. 3B), suggesting that LkhA plays an essential role in sexual development in *A. flavus*.

### Role of LkhA in stress response

**Fig. 4.**
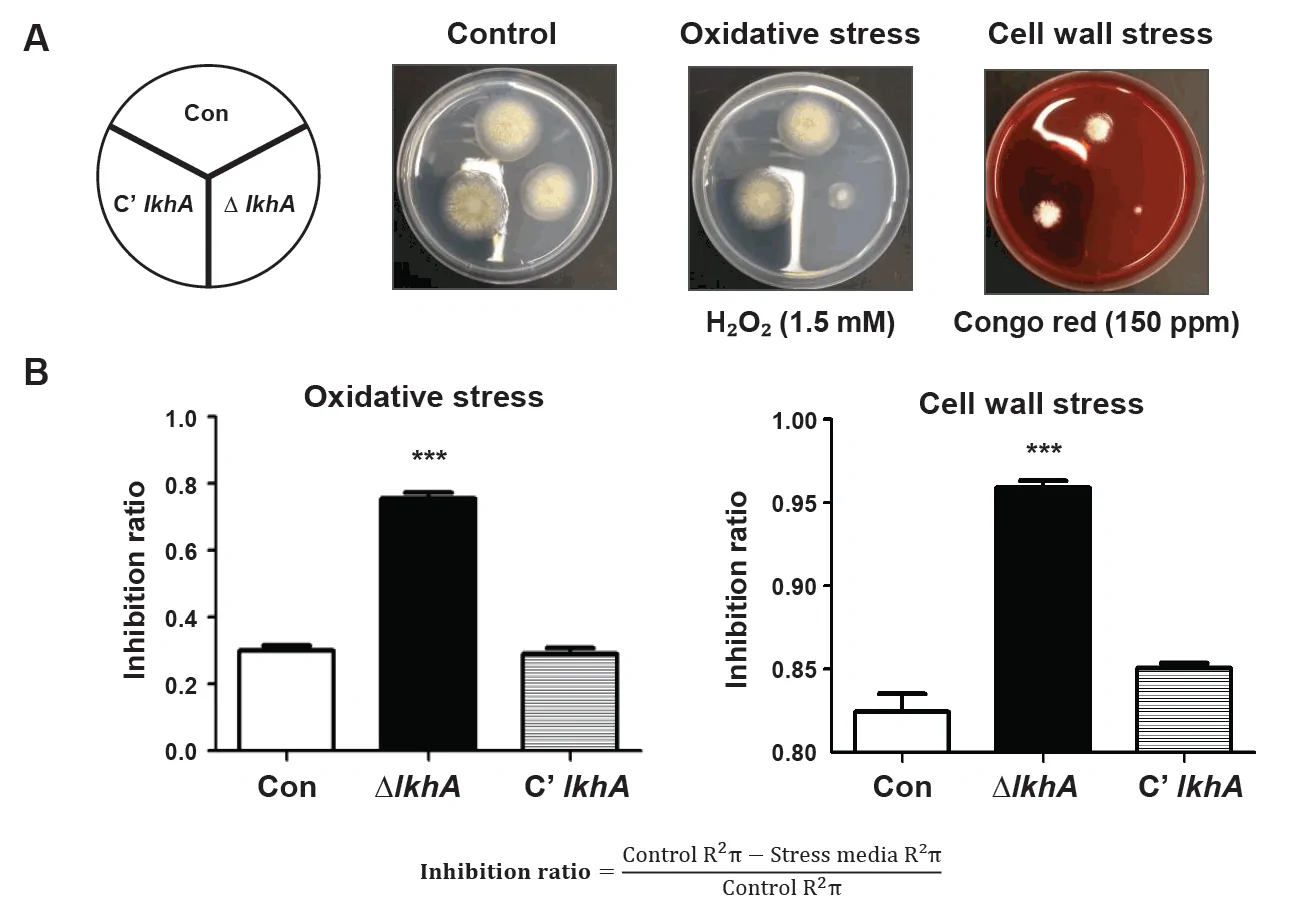
Characterization of roles of LkhA in stress response. (A) Photographs of control, *lkhA* deletion mutant, and *lkhA* complemented strains grown at 37°C for 3 days in MMYE medium supplemented with hydrogen peroxide (oxidative stressor) or Congo red (cell wall stressor). (B) Quantitative data of inhibition ratio, and formula for calculating inhibition ratio (^***^p ≤ 0.001).

Studies have revealed that the LAMMER kinases Kns1 in *S. cerevisiae* and Lkh1 in *Schizosaccharomyces pombe* are important for various stress responses (Kang et al., 2007; Park et al., 2003; Park and Park, 2011). To assess the role of LkhA in *A. flavus*, we inoculated control and Δ*lkhA* strains onto media containing H_2_O_2_ or Congo red. As shown in Fig. 4, the growth of the Δ*lkhA* strain was significantly reduced under oxidative or cell wall stress conditions. Under osmotic stress conditions, no significant change was noted in the growth of the Δ*lkhA* strain compared with that of the control or *C' lkhA* strain (data not shown). These results suggest that LkhA is required for fungal response to a cell wall-perturbing agent or an oxidative stressor.

### Role of LkhA in aflatoxin B1 production

**Fig. 5.**
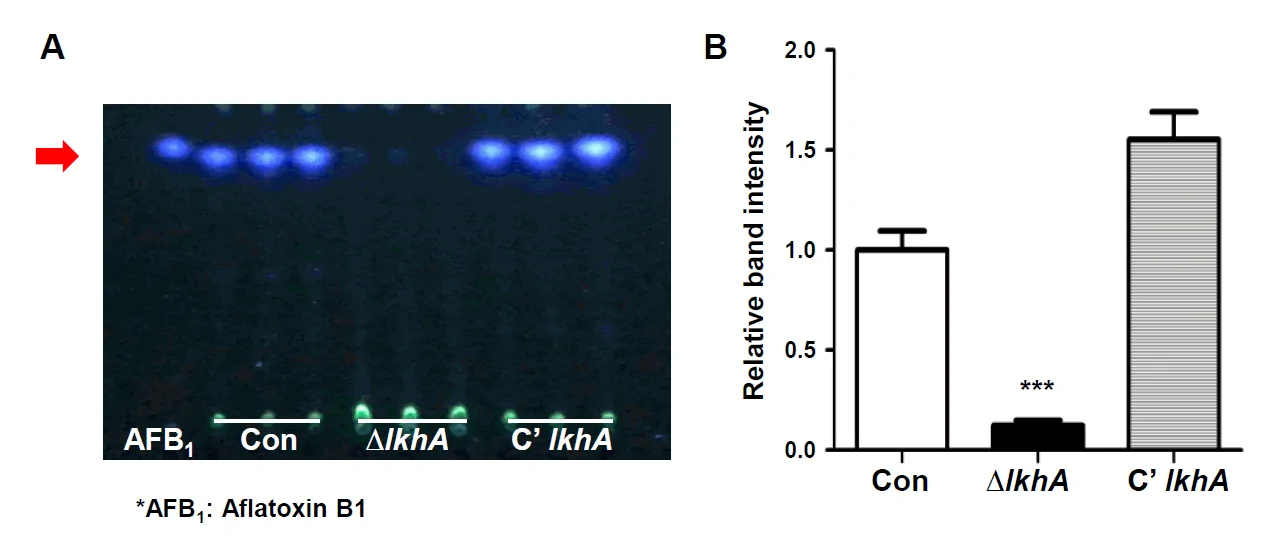
Roles of LkhA in aflatoxin B1 production. (A) Photograph of aflatoxin B1 isolated by thin-layer chromatography from control, *lkhA* deletion mutant, and *lkhA* complemented strains. Aflatoxin B1 was extracted from strains grown for 7 days in CM. Aflatoxin B1 was used as a standard substance. (B) Band intensity of aflatoxin B1 assessed using ImageJ software (^***^p ≤ 0.001).

To assess the role of LkhA in aflatoxin B1 production, we cultured control, Δ*lkhA*, and *C' lkhA* strains in CM for 10 days. Subsequently, we extracted aflatoxin B1 from each cultured sample and quantified its amount using TLC. As shown in Fig. 5, the production of aflatoxin B1 was significantly lower in the Δ*lkhA* strain than in the control strain. This suggests that LkhA plays an important role in aflatoxin B1 production in *A. flavus*.

### Role of LkhA in pathogenicity

**Fig. 6.**
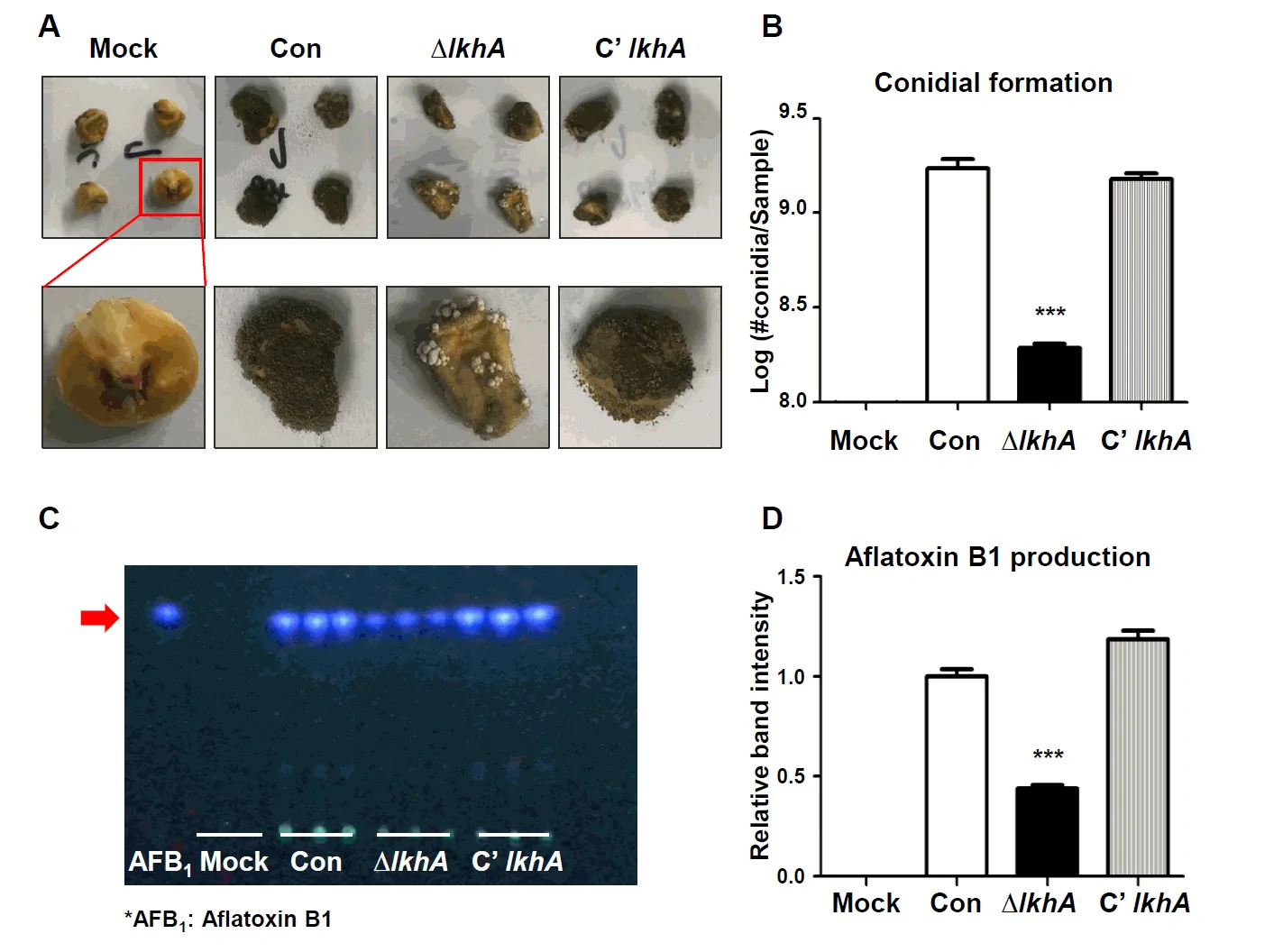
Roles of LkhA in plant pathogenicity. (A) Photograph of corn kernels inoculated with control, *lkhA* deletion mutant, and *lkhA* complemented strains. (B) Quantitative data of the number of conidia in *A. flavus* inoculated on corn. (C) Photograph of aflatoxin B1 isolated from each strain by TLC. (D) Band intensity of aflatoxin B1 separated by TLC using ImageJ software (^***^p ≤ 0.001).

To determine whether LkhA from *A. flavus* is involved in pathogenicity, we inoculated control, Δ*lkhA*, and *C' lkhA* strains onto corn. We then assessed conidial formation and aflatoxin B1 production (Fig. 6A). The Δ*lkhA* strain exhibited lower conidial formation than the control strain (Fig. 6B). Moreover, the Δ*lkhA* strain exhibited lower aflatoxin B1 production than the control and *C' lkhA* strains (Fig. 6C and 6D). These results suggest that LkhA is required for proper plant pathogenicity.

## Discussion

LAMMER kinases are conserved in most eukaryotes and are essential for appropriate regulation of growth, development, and stress responses (Lim and Park, 2019). Studies have revealed that LAMMER kinases play a key role in fungal development (Lim and Park, 2019). In *A. nidulans*, the Δ*lkhA* strain was found to form smaller colonies and produce fewer asexual spores than the wild type. The Δ*lkhA* strain also had an abnormal structure (Kang et al., 2013). Moreover, the Δ*lkhA* strain exhibited reduced colony growth and reduced number of conidia (Lim et al., 2021). In the present study, the deletion of *lkhA* affected fungal growth, conidial production, and conidiophore structure (Fig. 2). These results suggest that LkhA is a key component for regulating fungal growth and asexual development. Further analyses revealed that the absence of *lkhA* lowered the mRNA expression of *brlA*, a key initiator of conidiation, in the three *Aspergillus* strains. The mRNA expression of *abaA* also decreased in *A. nidulans* and *A. flavus*. Taken together, we propose that LkhA regulates conidiogenesis through the regulation of BrlA–AbaA–WetA.

We also found that the production of sexual reproductive structures known as sclerotia was significantly reduced or absent in the Δ*lkhA* strain (Fig. 3). In *A. nidulans*, the Δ*lkhA* strain was found to produce immature cleistothecia containing few ascospores (Kang et al., 2013). A similar phenotype was confirmed in *A. fumigatus*, which produced cleistothecia but with a significantly smaller amount of ascospores (Lim et al., 2021). In *A. nidulans* and *A. fumigatus*, LkhA was confirmed to play an important role in the maturation of cleistothecia; however, in *A. flavus*, it was hardly able to form sclerotia. Hence, LkhA can be predicted to be involved from the early stage of sexual development.

Fungal LAMMER kinases are required for stress tolerance (Lim and Park, 2019). However, LAMMER kinases respond differently to various stresses depending on the fungal species. For example, in *S. pombe*, Lkh1 is required for oxidative stress tolerance. However, in *A. fumigatus*, LkhA is not involved in oxidative stress tolerance (Kang et al., 2007; Lim et al., 2021). In *A. nidulans* and *Candida albicans*, LkhA and Kns1, respectively, are required for proper cell wall biosynthesis (Lim and Park, 2019). In this study, we found that fungal growth was defective under oxidative and cell wall stress conditions (Fig. 4). However, no growth defects were noted under osmotic stress conditions (data not shown). Overall, these findings suggest that the LAMMER kinases play a vital role in stress response; however, their role varies among fungal species.

*A. flavus* not only produces mycotoxins but also acts as a plant and human pathogen (Klich, 2007). The production of aflatoxins by *A. flavus* is influenced by environmental conditions, with oxidative stress being particularly linked to aflatoxin biosynthesis (Sweany et al., 2022). The Δ*lkhA* strain exhibited significantly reduced aflatoxin production in CM (Fig. 5); however, aflatoxin production was only partially reduced in the kernel model (Fig. 6). Hence, these changes in aflatoxin B1 production may be attributed to differences in culture conditions. Aflatoxin B1 produced by *A. flavus* is classified as a group 1 carcinogen that can cause hepatocellular carcinoma (Rushing and Selim, 2019). Aflatoxins produced by *A. flavus* in various crops, such as corn and maize, cause economic losses; however, their specific impact on plant pathogenicity has not been studied so far (Sweany et al., 2022). We noted a decrease in aflatoxin B1 production due to the deletion of *lkhA* in CM and the kernel model. These results support that LkhA can affect human pathogenicity; however, further research is warranted to determine its role in plant pathogenicity.

The role of LAMMER kinases has been studied in several fungi (Lim and Park, 2019). In the plant pathogenic fungus *Magnaporthe oryzae* (Li et al., 2022), the *Δkns1* mutant strain exhibited reduced asexual spore production. Moreover, no spores were formed on barley leaves, indicating a decrease in pathogenicity (Li et al., 2022). In *A. flavus*, the Δ*lkhA* strain exhibited reduced production of conidia in the kernel assay (Fig. 6), confirming that LkhA plays a role in plant pathogenicity. Studies have also been conducted on human pathogenic fungi, including *C. albicans*, *A. fumigatus*, and *Cryptococcus neoformans*. In *C. albicans*, the deletion of KNS1 affected adhesion ability in a zebrafish embryo model; however, it did not influence embryo hatching (Lim et al., 2020). In *A. fumigatus*, the Δ*lkhA* mutant strain exhibited a decreased mortality rate in zebrafish larvae (Lim et al., 2021). In *C. neoformans*, the Δ*lkhA* mutant strain was avirulent in a systemic murine model (Kwon et al., 2024). Although the role of LkhA in human or animal pathogenicity has not been studied in *A. flavus*, the decrease in aflatoxin B1, a key virulence factor, suggests that LkhA influences human pathogenicity. Further research is warranted to confirm its pathogenicity in humans or animals.

Overall, our study confirmed that LkhA plays an important role in development, stress tolerance, aflatoxin production, and pathogenicity in *A. flavus*. To our knowledge, this is the first study to confirm the roles of LkhA in *A. flavus*. Our results will provide insights into the development of antifungal agents for *A. flavus*, which infects crops, causes economic losses, and harms humans.

## Figures and Tables

**Table 1.** *Aspergillus flavus* strains used in this study

Strain	Relevant genotype	Reference
NRRL 3357	*A. flavus* wild type	ATCC collection
NRRL 3357.5	*pyrG^−^*	He et al. (2007)
TTJ6.1	*pyrG^−^*; *ΔpyrG*::*AfupyrG^+^*	Lim et al. (2019)
TJSH4.1–3	*pyrG^−^*; *ΔlkhA*::*AfupyrG^+^*	This study
TJSH5.1 and 2	*pyrG^−^*; *lkhA(p)*::*lkhA*::*FLAG_4x_*::*ptrA*; *ΔlkhA*::*AfupyrG^+^*	This study
		

**Table 2.** Primers used in this study

Name	Sequence (5′→3′)^a^	Purpose
OHS1542	CCTGGTCTTTGGTTTGGTACACC	*AfupyrG* Maker_F
OHS1543	CGACTGGCAGGAGATGATCC	*AfupyrG* Maker_R
OHS2349	CTACACTTCGATGAGCCTTGC	5′ *AfllkhA* DF
OHS2350	*GGCTTTGGCCTGTATCATGACTTCA* GCAGTGACCGTCGTACTCTAC	3′ *AfllkhA* with *AfupyrG* tail
OHS2351	*TTTGGTGACGACAATACCTCCCGAC* GTCGTGTTGATCTCAAGTGGC	5′ *AfllkhA* with *AfupyrG* tail
OHS2352	CAGTGAGAACTCGTGCAGG	3′ *AfllkhA* DR
OHS2353	CTCTCGGTATTACTGACTACGACG	5′ *AfllkhA* NF
OHS2354	GTTCTGCACGACAGTGCAT	3′ *AfllkhA* NR
OHS2355	CATCATCAGCAGCACTACGG	5′ *AfllkhA* RT_F
OHS2356	CGTAGGGATATGTGGTGGCA	3′ *AfllkhA* RT_R
OHS2565	aatt GCGGCCGC CCTACCTCAGATCGACATTGCA	5′ *AfllkhA* NotI_F
OHS2405	aatt GCGGCCGC GGTGTTGCGCTGCAACTG	3′ *AfllkhA* NotI_R
OHS407	GATATGTCGCCACACTGGAC	*AflbrlA* RT_F
OHS408	CTGTATTCGCGGCTATTCGG	*AflbrlA* RT_R
OHS409	CTTCCGCACCTTAACAGCAG	*AflabaA* RT_F
OHS410	GTTTGCCGGAATTGCCAAAG	*AflabaA* RT_R
OHS405	TATGTCGGTGATGAGGCACA	*Aflactin* RT_F
OHS406	AACACGGAGCTCGTTGTAGA	*Aflactin* RT*_R*

a Tail sequences are shown in italics. Restriction enzyme sites are presented in bold.
